# Diagnosis and Treatment of Hypertension Among People Aged 45 Years and Over in India: A Sub-national Analysis of the Variation in Performance of Indian States

**DOI:** 10.3389/fpubh.2021.766458

**Published:** 2021-10-29

**Authors:** Mrigesh Bhatia, Manish Kumar, Priyanka Dixit, Laxmi Kant Dwivedi

**Affiliations:** ^1^Department of Health Policy, London School of Economics, London, United Kingdom; ^2^Department of Mathematical Demography & Statistics, International Institute for Population Sciences, Mumbai, India; ^3^School of Health Systems Studies, Tata Institute of Social Sciences, Mumbai, India

**Keywords:** hypertension, awareness, diagnosis, treatment, variation in performance, India

## Abstract

**Introduction:** Cardiovascular disease (CVD) is the single largest contributor to non-communicable disease (NCD) deaths, with hypertension contributing to a significant proportion of these deaths. This study aims to provide estimates of the prevalence, awareness, treatment and control of hypertension at sub-national levels in India and identifies well and under-performing states with respect to the diagnosis and treatment of hypertension.

**Methods:** The study utilises data from the Longitudinal Study of Ageing in India (LASI), a nationally representative survey of more than 72,000 individuals. Age-sex adjusted prevalence rates of self-reported hypertension was calculated using the direct standardisation method. Multivariable logistic regression was performed to assess the association of self-reported hypertension with the various individual co-morbidity, lifestyle, and household factors. Self-reported prevalence was compared with an objective measure of hypertension for each state, and funnel plots were constructed to assess the performance of states.

**Results:** Our findings suggest that the overall prevalence of age-sex adjusted self-reported hypertension was 25.8% in India with significant variation among states. Results based on logistic regression confirm that those individuals who are elderly, obese, belong to a higher socio-economic group and have associated co-morbidities are at increased odds of reporting hypertension. Overall, 4 out of 10 adults over 45 years of age in India are not aware of their hypertensive condition, and of those who are aware, 73% are currently taking medication, and only 10% of these have their hypertension under control. Based on the performance, states were classified into high and low performing categories. States with an increased proportion of population below the poverty line had significantly lower performance with respect to the diagnosis of hypertension, whereas states with higher literacy rates and greater availability of specialist doctors at community health centres (CHCs) had significantly better performance with respect to treatment-seeking behaviour.

**Conclusion:** The findings of this study and its policy implications are discussed. Based on state performance, strategies are proposed in terms selective targeting vs. population-based strategies. High impact states and sub-groups are identified where intense efforts are needed to tackle the growing menace of hypertension in India.

## Introduction

Non-communicable diseases (NCDs) are a leading cause of mortality worldwide and disproportionately affect low and middle income countries. According to the World Health Organisation (WHO), NCDs account for 71% of all deaths globally, and 77% of these deaths occur in low and middle income countries (1). Cardiovascular disease (CVD) is the single largest contributor with over 44% of all NCD deaths (1). Not only are CVDs rising rapidly in low and middle income countries, but unlike the west, these diseases are affecting younger age groups that are economically productive. For example, 30% of NCD-related deaths in low income countries occur under the age of 60, whereas in high income countries, the proportion is only 13% (2). India is no exception to this trend. According to the Global Burden of Disease (GBD) Study, disability adjusted life years (DALYs) as a result of NCDs have increased from 29.2% in 1990 to 57.9% in 2019 (3).

NCDs are not only a major burden on already weak public health care systems further weakened by COVID, but also contribute significantly to financial hardship in households in many low and middle income countries. The economic burden of NCDs is enormous. A study estimated that the economic loss due to NCDs over the next two decades would represent 75% of global Gross Domestic Product (GDP) (4). It is therefore not surprising to note efforts by the global community to reduce the burden of NCDs. For example, the United Nations (UN) sustainable development goal 3 (SDGs) aims to reduce premature mortality from NCDs by a third by 2030 (5).

Hypertension (HT) is one of the commonest NCD, and a major public health concern accounting for 19% of all NCD deaths globally (1). In South Asia, HT is estimated to be the third leading cause of death and disability, after household air pollution and tobacco smoking (6). In addition, it is an independent risk factor for coronary heart disease; the asymptomatic nature of HT contributes to a lack of awareness of this condition, thus being labelled a “silent killer disease.” If undiagnosed or uncontrolled, HT can significantly contribute to unnecessary death and disability due to coronary heart disease. Hence, it is crucial that the basic principle of levels of prevention in public health is adhered to, including early diagnosis and prompt treatment.

India is the second most populous nation, contributing to 18% of the world's population (7), and is one of the fastest growing economies in the world. The country ranked 131 among 188 countries in the SDG progress indicators (8), with widespread diversity among regions and states of India. Health in India is a state subject, in that the responsibility of financing and delivering health care lies with the respective states. However, there is considerable variation among the states in terms of their population coverage, human development index (HDI), the level of demographic and epidemiological transition taking place, and health system capacity including supply side constraints, all of which have an impact on the prevalence of disease and the quality of health service that the state is able to provide to its population. For example, states like Kerala and Goa, with an HDI of over 7.5, experience health indicators comparable to Sri Lanka and China, whereas states like Jharkhand, Uttar Pradesh and Bihar with an HDI below 6 experience health indicators comparable to Kenya, Cambodia and the Republic of Congo (9). This relationship is reversed in the case of NCDs and HT, where more developed states with a higher HDI, and urbanisation experience higher HT prevalence rates (10).

There have been number of studies reporting on the prevalence of HT across various geographical regions in India (11–16) and occupational status groups (17–19). According to a recent study based on a national-level blood pressure survey, the prevalence of HT among individuals aged 18+ and 65+ years was nearly 30 and 52%, respectively (20). Moreover, a meta-analysis based on 142 communities-based studies in India observed significant differences in the prevalence of HT across the Indian regions, where HT prevalence in rural regions varies between 14.5% (North) and 31.7% (East), while in urban regions it varies between 28.8% (North) and 35.8% (West) (21). This region-wide variation demands updated state-level estimates for the prevalence, awareness, treatment and control of HT based on nationally representative data for older Indian adults. In addition, although there are few studies reporting on the prevalence of HT in which individual socio-economic characteristics were analysed, there are hardly any studies that have analysed the variation in state performance with respect to the diagnosis and treatment of HT on a nationally representative survey.

Given the decentralised health care system in India and considerable variation in access to health care, varying levels of access to government health facilities and high out of pocket payments, a disaggregate analysis providing estimates of performance of states with respect to the diagnosis and treatment of HT would benefit sub-national policy makers to identify and target priority states and sub-groups within the states where intense efforts are needed to effectively plan interventions and strategies related to tackling the burden of HT. Such research is all the more necessary, as a study over two decades has concluded that, in spite of a significant increase in the prevalence of HT in India, there has been no improvement in the management of HT during this time (22).

This study therefore aims to identify the characteristics of those who have been diagnosed as hypertensive and are taking treatment, and to assess the performance of states with respect to diagnosis and treatment of HT. Through the use of maps, logistic regression and funnel plots, in addition to undertaking a disaggregate analysis at the sub-national level, the present study contributes to the existing literature by providing current estimates of the prevalence, awareness, treatment and control of HT, and identifies well- and under-performing states with respect to the diagnosis and treatment of HT. Furthermore, the study investigates the possible determinants of the prevalence and treatment of HT among older adults aged 45 years and above and the causes for the variation in performance by linking it to state development, supply side constraints, public health system capacities and the role of the private sector. Finally, by identifying high impact states and sub-groups, this paper makes policy recommendations to ensure government policies, programmes and limited resources are better targeted to key states and high risk groups where intense efforts are needed in order to reduce the mortality and morbidity associated with HT in India.

## Materials and Methods

### Data Source and Study Population

We used data from the Longitudinal Ageing Survey of India (LASI, 2017–18) which is a national representative survey of over 72,000 older adults aged 45 years and above (including spouses irrespective of their age) across all states and UTs in India, except Sikkim. The main objective of the LASI survey was to provide scientific evidence on demographics, household economic status, chronic health conditions, functional and mental health, biomarkers, health care utilisation, work and employment, etc. LASI adopted a multistage stratified area probability cluster sampling design to arrive at the eventual units of observations: a three-stage sampling design in rural areas and a four-stage sampling design in urban areas. The detailed methodology, with the complete information on the survey design and data collection, was published in the survey report (23). The present study was based on the eligible older adults aged 45 years and above, and the effective sample size was 65,562.

### Measures

Self-reported HT was assessed by asking the question, “*Has any health professional ever told you that you have HT or high blood pressure?*” The participants who responded “Yes” to this question were considered hypertensive. Only self-identified hypertensive participants were further asked about their treatment-seeking behaviour: “*In order to control your blood pressure or HT, are you currently taking any medication?*” In the biomarker measurements section, LASI also provides the blood pressure measurements of older adults. HT was defined as the average of the last two readings of systolic blood pressure (SBP) ≥ 140 mmHg or/and diastolic blood pressure (DBP) ≥ 90 mmHg. The prevalence of 'overall HT' is defined as the proportion of hypertensive older adults either by self-reported or biometric measurement. Controlled HT is defined as SBP < 140 mmHg or DBP <90 mmHg and currently taking anti-hypertensive medication (24).

### Covariates

#### Socio-Demographic Variables

Various demographic variables such as gender (male, female), age (45–54, 55–64, 65–74, or 75+ years), education (no education, primary, secondary, or higher), working status (never worked, currently working or not currently working), and marital status (currently married, widowed or divorced/separated/deserted) were included in the analysis. LASI collected information from households about their spending on food (a reference period of 7 days) and non-food items (reference periods of 30 and 365 days). After standardising the food and non-food expenditure to a 30-day reference period, the monthly per capita consumption expenditure (MPCE) was computed and used as the summary measure of consumption: poorest, poorer, middle, richer, and richest. Various other household factors, including caste (scheduled tribe, scheduled caste, other backward class, or other), religion (Hindu, Muslim, Christian, or other), and place of residence (rural or urban), and region (North, Central, East, Northeast, West, and South) of residence were included in the analysis.

#### Health Status

Body mass index (BMI) was recoded as underweight (<18.5), normal (18.5–24.9), overweight (25–29.9) or obese (30 and above); we have combined overweight and obese for analytical purposes. We have included three self-reported chronic diseases (diabetes, arthritis and stroke) diagnosed by a health professional. Functional health was assessed by basic and instrumental activities of daily living (ADLs). Six basic ADLs (BADLs) include dressing, indoor mobility, bathing, eating difficulties, getting in or out of bed and using the toilet, and seven instrumental ADLs (IADLs) include food preparation, shopping for groceries, taking medication, making telephone calls, doing work around the house or garden, ability to handle finances and getting around or finding an address in unfamiliar places. We created two variables for assessing the functional limitations: difficulty in ADLs (at least one difficulty in six BADLs) and difficulty in IADLs (at least one difficulty in seven IADLs).

#### Lifestyle Behaviours

In LASI, the participants were asked about their tobacco use status (smoking and smokeless). Based on the information, we have classified the participants as: never, former or current tobacco users. Alcohol drinking status was assessed with a yes/no question. To assess the level of physical activities among older adults, LASI collected information on moderate (*washing clothes, cleaning the house, fetching water, drawing water from a well, gardening, walking at a moderate pace, bicycling at a regular pace, and floor or stretching exercises*) and vigorous (*swimming, running or jogging, going to health centre/gym, cycling, digging with a spade or shovel, heavy lifting, chopping, farm work, fast bicycling, and cycling with a load*) physical activities. The possible responses for moderate and vigorous physical activities were: every day, more than once a week, once a week, one to three times per month and hardly ever or never. Based on these responses, we classified the respondent as physically active (more than once a week) and physically inactive (engagement of once a week or less often) for both moderate and vigorous activities.

In addition, macro-level secondary data, which may affect self-reported HT prevalence and treatment among Indian older adults, was collected for different covariates to explain state level variations in performance. Information about the state-wise HDI and percentage of persons below the poverty line (BPL) was obtained from Indiastat.com. Data for the variables, including doctors available at primary health centres (PHCs), specialists available at community health centres (CHCs), and per capita health expenditure were obtained from India's National Health Profile report (25). Information about state-wise literacy rates was obtained from the Census of India (2011). Moreover, the Longitudinal Ageing Survey in India report (23) was used to derive the data for state-wise out-of-pocket expenditure (OOPE).

### Statistical Analysis

We calculated the age-sex adjusted prevalence rates of self-reported HT and treatment of HT for all states and Union Territories (UTs) using the direct standardisation method. The age-sex structure of the national population from Census 2011 was used as the reference population for carrying out the standardisation. We assessed the diagnosis-based performance of all the states defined as the ratio of older adults who are aware of their HT status and overall HT. We further constructed funnel plots to observe the variation in diagnosis-based performance, HT treatment, and controlled HT between states. The national average of diagnosis-based performance, HT treatment, and controlled HT (indicated by a solid line parallel to the x-axis) was used as the baseline reference. The 95 and 99% confidence bands were also created on the funnel plots. We used multivariable logistic regression to assess the association of self-reported HT and treatment of HT with the various individual (i.e., age, education, working status and marital status), morbidities (i.e., diabetes, stroke and arthritis), lifestyle (i.e., smoking status, chewing tobacco, drinking alcohol, moderate and vigorous activities) and household (i.e., MPCE quintile, religion, caste and residence) factors. Finally, a regression model using select state level covariates was performed to explain the variation in state performance with respect to the diagnosis and treatment of HT.

## Results

As observed in Table 1, the prevalence of unadjusted self-reported HT subjects had a 1.6 percentage point greater prevalence than adjusted self-reported HT subjects (27.4 vs. 25.8%). The results indicate that the sex and age-adjusted prevalence of self-reported HT varied greatly between states and UTs, with a prevalence of about 16% in Chhattisgarh, Nagaland, and Dadra and Nagar Haveli to prevalence of 41% in Punjab, Goa and Andaman and Nicobar Islands. Interestingly, the prevalence of self-reported adjusted HT was highest in the states belonging to the northern region, namely Jammu and Kashmir (37.7%), Chandigarh (37.9%), Haryana (37%), Delhi (35.8%), and Kerala (36.6%) from the southern region. On the contrary, self-reported prevalence of HT was relatively low in states belonging to the central region such as Uttar Pradesh (19.5%), Madhya Pradesh (19.3%) and Odisha (19.2%) from the eastern part of India.

**Table 1:** Unadjusted and age-sex adjusted prevalence of self-reported and its treatment, India, LASI, 2017–18.

	**Unadjusted % (95% CI)**		**Adjusted % (95% CI)**	
	**Self-reported HT**	**Taking treatment for HT**	**Self-reported HT**	**Taking treatment for HT**
**State/UT**				
Jammu and Kashmir	40.6 (38.1, 43.1)	85.3 (82.4, 88.2)	37.7 (34.6, 40.9)	82.5 (76.9, 88.1)
Himachal Pradesh	32.9 (30.3, 35.5)	63.7 (59.0, 68.5)	30.4 (27.4, 33.4)	60.1 (53.6, 66.6)
Punjab	42.8 (40.6, 45.0)	73.9 (71.0, 76.9)	40.7 (38.0, 43.4)	73.0 (68.8, 77.2)
Chandigarh	39.6 (36.4, 42.7)	80.7 (76.7, 84.7)	37.9 (34.4, 41.4)	76.8 (71.5, 82.1)
Uttarakhand	26.6 (24.2, 29.1)	56.6 (51.3, 61.8)	25.2 (22.5, 27.9)	56.0 (49.3, 62.7)
Haryana	38.4 (36.2, 40.7)	55.2 (51.4, 59.0)	37.0 (34.4, 39.5)	53.0 (48.3, 57.7)
Delhi	35.8 (33.0, 38.5)	68.1 (63.5, 72.8)	35.8 (32.2, 39.5)	63.4 (57.2, 69.5)
Rajasthan	27.3 (25.4, 29.2)	59.7 (55.7, 63.7)	25.6 (23.5, 27.7)	57.4 (52.1, 62.7)
Uttar Pradesh	20.0 (18.8, 21.2)	57.5 (54.2, 60.7)	19.5 (18.1, 20.9)	56.0 (52.0, 60.1)
Bihar	25.1 (23.6, 26.6)	49.2 (45.8, 52.6)	23.7 (21.6, 25.8)	44.1 (39.4, 48.8)
Arunachal Pradesh	22.6 (20.0, 25.2)	31.7 (25.2, 38.2)	23.0 (19.2, 26.7)	34.0 (25.7, 42.4)
Nagaland	15.8 (13.8, 17.9)	60.5 (52.8, 68.2)	15.5 (10.2, 20.8)	61.5 (48.5, 74.6)
Manipur	28.7 (26.2, 31.2)	69.9 (64.9, 74.8)	27.1 (23.8, 30.3)	64.6 (57.5, 71.7)
Mizoram	24.0 (21.5, 26.5)	43.7 (37.8, 49.6)	21.8 (18.7, 25.0)	40.6 (32.8, 48.3)
Tripura	30.4 (27.6, 33.2)	68.0 (62.8, 73.2)	29.4 (26.5, 32.4)	66.2 (60.2, 72.1)
Meghalaya	25.9 (23.0, 28.8)	78.1 (72.6, 83.6)	23.3 (20.3, 26.3)	73.1 (64.4, 81.9)
Assam	31.1 (29.1, 33.1)	64.7 (60.9, 68.5)	30.5 (28.4, 32.7)	61.6 (57.2, 65.9)
West Bengal	29.6 (28.0, 31.1)	74.6 (72.1, 77.2)	28.0 (26.0, 30.1)	72.2 (68.0, 76.3)
Jharkhand	21.7 (20.0, 23.4)	64.1 (59.8, 68.4)	20.2 (18.4, 22.0)	61.7 (56.4, 67.0)
Odisha	20.4 (18.9, 21.9)	67.7 (63.8, 71.6)	19.2 (17.6, 20.7)	64.5 (59.6, 69.4)
Chhattisgarh	16.5 (14.8, 18.2)	68.8 (63.6, 74.1)	16.3 (14.5, 18.1)	66.0 (60.1, 71.9)
Madhya Pradesh	20.0 (18.5, 21.5)	64.2 (60.2, 68.2)	19.3 (17.0, 21.6)	58.3 (52.1, 64.6)
Gujarat	25.7 (23.9, 27.6)	69.2 (65.2, 73.1)	24.2 (22.1, 26.3)	63.9 (58.5, 69.4)
Daman and Diu	32.9 (29.9, 36.0)	79.8 (75.1, 84.4)	31.2 (27.4, 35.0)	75.8 (68.6, 83.0)
Dadra and Nagar Haveli	17.0 (14.7, 19.3)	69.4 (61.8, 76.9)	16.9 (14.1, 19.7)	68.9 (60.4, 77.5)
Maharashtra	28.9 (27.4, 30.3)	86.4 (84.4, 88.5)	26.0 (24.3, 27.8)	82.8 (79.2, 86.4)
Andhra Pradesh	34.9 (33.0, 36.9)	88.3 (86.2, 90.5)	33.3 (31.3, 35.3)	86.6 (83.8, 89.4)
Karnataka	32.7 (30.7, 34.7)	91.7 (89.3, 94.1)	31.1 (23.3, 38.9)	89.5 (82.2, 96.9)
Goa	44.1 (41.4, 46.8)	94.7 (92.8, 96.6)	40.8 (37.4, 44.1)	93.0 (89.7, 96.3)
Lakshadweep	35.5 (32.6, 38.4)	76.7 (72.6, 80.8)	33.3 (29.6, 36.9)	74.3 (68.0, 80.7)
Kerala	41.0 (39.0, 43.1)	87.6 (85.5, 89.7)	36.6 (33.7, 39.6)	81.0 (76.2, 85.8)
Tamil Nadu	26.3 (24.8, 27.9)	76.7 (74.0, 79.4)	24.6 (22.7, 26.4)	75.1 (71.1, 79.1)
Puducherry	32.7 (30.1, 35.3)	87.8 (84.9, 90.8)	29.8 (26.8, 32.7)	84.7 (79.8, 89.6)
Andaman and Nicobar Islands	41.2 (38.3, 44.1)	78.8 (75.0, 82.6)	40.4 (36.2, 44.6)	76.4 (70.7, 82.1)
Telangana	31.0 (29.1, 32.9)	87.6 (85.2, 90.0)	28.7 (26.7, 30.8)	87.7 (84.9, 90.6)
**India**	**27.4 (27.1, 27.7)**	**73.0 (72.4, 73.6)**	**25.8 (24.9, 26.7)**	**70.1 (68.2, 72.0)**

*HT, hypertension*.

The unadjusted current treatment seeking for HT was 2.9 percentage points greater than adjusted treatment seeking (73 vs. 70.1%). Adjusted treatment seeking among those aged 45 years and above varied significantly across the states and UTs in India, from about 34% in Arunachal Pradesh to 93% in Goa. It is important to note that Goa was one of the states where adjusted self-reported HT was also highest among all the states. Mostly high treatment seeking was observed in southern states like Karnataka (89.5%), Telangana (87.7%), Andhra Pradesh (86.6%), Puducherry (84.7%) and Kerala (81%) and was low in Mizoram (40%), and Bihar (44%).

Table 2 shows the adjusted odds ratios (AOR) for self-reported HT separately for men and women. A range of individual variables, presence of co-morbidities, lifestyle factors and household factors were included in the model. The results show that increasing age positively affected HT in both genders, and currently working men and women were less likely to report HT compared to individuals who never worked. Compared with individuals with a normal BMI, individuals who were overweight or obese were more likely to suffer with HT [AOR (95%CI): ranging from 1.68 (1.56–1.80) in men to 1.79 (1.69–1.90) in women]. All the morbidity-related factors like presence of diabetes, stroke, arthritis and difficulty in ADL and IADL were positively related to HT prevalence in both men and women. Among the lifestyle factors, if women were moderately active and men were vigorously active, then they had less chance of having HT relative to inactive individuals. Household characteristics were also significantly associated with the risk of self-reported HT. Individuals who belonged to poorer to the richest households (compared with the poorest households), or belonged to other religion (compared with Hindu) and from an urban area (compared with rural) were associated with an increased risk of HT. However, the AORs of the individual from scheduled tribe (compared to scheduled caste) and from the central and western regions (compared with the northern region) were significantly less likely to report HT.

**Table 2:** Logistic regression results for self-reported hypertension among older adults, India, LASI, 2017–18.

	**AOR (Overall)**	**95% CI**	**AOR (Men)**	**95% CI**	**AOR (Women)**	**95% CI**
**Individual factors**						
**Age groups**						
45–54	Ref.		Ref.		Ref.	
55–64	1.39^***^	(1.32, 1.46)	1.41^***^	(1.30, 1.53)	1.41^***^	(1.32, 1.50)
65–74	1.76^***^	(1.66, 1.87)	1.88^***^	(1.71, 2.07)	1.81^***^	(1.68, 1.96)
75+	1.80^***^	(1.66, 1.95)	2.07^***^	(1.82, 2.34)	1.85^***^	(1.66, 2.07)
**Education level**						
No education	Ref.		Ref.		Ref.	
Primary	1.13^***^	(1.07, 1.19)	1.18^***^	(1.09, 1.29)	1.25^***^	(1.16, 1.34)
Secondary	1.06	(0.99, 1.12)	1.25^***^	(1.14, 1.37)	1.09	(1.00, 1.19)
Higher	1.11*	(1.02, 1.20)	1.42^***^	(1.27, 1.59)	0.98	(0.86, 1.11)
**Working status**						
Never worked	Ref.		Ref.		Ref.	
Currently working	0.69^***^	(0.66, 0.73)	0.81*	(0.69, 0.95)	0.79^***^	(0.74, 0.85)
Not currently working	0.97	(0.92, 1.02)	1.13	(0.96, 1.33)	1.05	(0.98, 1.12)
**Marital status**						
Currently married	Ref.		Ref.		Ref.	
Widowed	1.29^***^	(1.23, 1.36)	0.97	(0.87, 1.08)	1.30^***^	(1.23, 1.39)
D/S/D/Others^a^	1.00	(0.89, 1.12)	0.85	(0.70, 1.02)	1.10	(0.95, 1.28)
**BMI categories**						
Normal	Ref.		Ref.		Ref.	
Underweight	0.64^***^	(0.60, 0.68)	0.63^***^	(0.58, 0.70)	0.64^***^	(0.59, 0.70)
Overweight/obese	1.77^***^	(1.69, 1.85)	1.68^***^	(1.56, 1.80)	1.79^***^	(1.69, 1.90)
**Morbidities**						
**Diabetes**						
No	Ref.		Ref.		Ref.	
Yes	3.51^***^	(3.32, 3.72)	3.61^***^	(3.33, 3.92)	3.46^***^	(3.20, 3.74)
**Stroke**						
No	Ref.		Ref.		Ref.	
Yes	3.32^***^	(2.85, 3.87)	3.62^***^	(2.97, 4.40)	3.03^***^	(2.37, 3.88)
**Arthritis**						
No	Ref.		Ref.		Ref.	
Yes	1.39^***^	(1.30, 1.49)	1.32^***^	(1.17, 1.48)	1.42^***^	(1.30, 1.54)
**Difficulty in ADL** ^b^						
No	Ref.		Ref.		Ref.	
Yes	1.20^***^	(1.13, 1.27)	1.27^***^	(1.15, 1.41)	1.16^***^	(1.07, 1.25)
**Difficulty in IADL** ^c^
No	Ref.		Ref.		Ref.	
Yes	1.17^***^	(1.11, 1.22)	1.15^***^	(1.06, 1.25)	1.13^***^	(1.06, 1.20)
**Lifestyle factors**						
**Moderate activities**						
Inactive	Ref.		Ref.		Ref.	
Active	0.97	(0.92, 1.01)	0.95	(0.89, 1.02)	0.90^***^	(0.85, 0.95)
**Vigorous activities**						
Inactive	Ref.		Ref.		Ref.	
Active	0.91^***^	(0.86, 0.96)	0.90^**^	(0.83, 0.96)	0.97	(0.90, 1.04)
**Smoking tobacco**						
Never	1.00	(1.00, 1.00)	1.00	(1.00, 1.00)	1.00	(1.00, 1.00)
Former	1.04	(0.95, 1.15)	1.06	(0.95, 1.18)	1.25	(0.99, 1.58)
Current	0.85^***^	(0.79, 0.91)	0.91*	(0.84, 0.98)	0.99	(0.85, 1.15)
**Chewing tobacco**						
Never	Ref.		Ref.		Ref.	
Former	1.05	(0.92, 1.19)	1.01	(0.86, 1.20)	1.13	(0.92, 1.40)
Current	0.93^**^	(0.88, 0.98)	0.89^**^	(0.82, 0.97)	1.03	(0.95, 1.12)
**Alcohol consumption**						
No	Ref.		Ref.		Ref.	
Yes	0.95	(0.89, 1.01)	1.06	(0.99, 1.14)	0.86	(0.74, 1.01)
**Household factors**						
**MPCE quintile**						
Poorest	Ref.		Ref.		Ref.	
Poorer	1.15^***^	(1.08, 1.23)	1.08	(0.98, 1.20)	1.19^***^	(1.09, 1.29)
Middle	1.25^***^	(1.17, 1.34)	1.16^**^	(1.04, 1.28)	1.30^***^	(1.20, 1.42)
Richer	1.37^***^	(1.28, 1.46)	1.30^***^	(1.17, 1.44)	1.39^***^	(1.28, 1.52)
Richest	1.42^***^	(1.33, 1.52)	1.36^***^	(1.23, 1.51)	1.43^***^	(1.30, 1.56)
**Religion**						
Hindu	Ref.		Ref.		Ref.	
Muslim	1.23^***^	(1.15, 1.31)	1.12^*^	(1.01, 1.24)	1.38^***^	(1.27, 1.50)
Christian	1.03	(0.94, 1.12)	1.00	(0.87, 1.15)	1.05	(0.94, 1.18)
Others^$^	1.26^***^	(1.15, 1.39)	1.27^***^	(1.10, 1.47)	1.34^***^	(1.18, 1.52)
**Caste**						
Scheduled caste	Ref.		Ref.		Ref.	
Scheduled tribe	0.76^***^	(0.70, 0.82)	0.78^***^	(0.69, 0.89)	0.74^***^	(0.66, 0.82)
OBC^#^	0.99	(0.93, 1.05)	0.98	(0.89, 1.07)	1.00	(0.92, 1.08)
Others	1.04	(0.97, 1.11)	1.07	(0.96, 1.18)	1.02	(0.93, 1.11)
**Place of residence**						
Rural	Ref.		Ref.		Ref.	
Urban	1.19^***^	(1.14, 1.25)	1.19^***^	(1.11, 1.28)	1.18^***^	(1.11, 1.26)
**Region**						
North	Ref.		Ref.		Ref.	
Central	0.67^***^	(0.62, 0.72)	0.69^***^	(0.61, 0.78)	0.66^***^	(0.60, 0.73)
East	0.84^***^	(0.79, 0.91)	0.92	(0.82, 1.03)	0.81^***^	(0.74, 0.88)
Northeast	1.00	(0.92, 1.10)	1.10	(0.96, 1.26)	0.87^*^	(0.78, 0.99)
West	0.77^***^	(0.71, 0.83)	0.83^**^	(0.74, 0.93)	0.71^***^	(0.64, 0.78)
South	0.84^***^	(0.79, 0.90)	0.96	(0.87, 1.06)	0.76^***^	(0.70, 0.83)

# *Other Backward Classes*.

$ *Includes Sikh, Buddhist/neo-Buddhist, Jain, Parsi/Zoroastrian and others*.

a *Divorced, separated, and deserted*.

b *Activities of daily living includes dressing, walking across a room, bathing, eating difficulties, getting in or out of bed and toilet use (any one or more)*.

c *Instrumental Activities of Daily Living (IADL) includes preparing a hot meal, shopping for groceries, making telephone calls, taking medications, doing work around the house or garden, managing money and getting around or finding address in unfamiliar place (any one or more)*.

* *p < 0.05*,

** *p < 0.01*,

*** *p < 0.001*.

*BMI, Body Mass Index; AOR, Adjusted Odds Ratio; CI, Confidence Interval*.

The AORs of current treatment seeking behaviour for HT using multivariable logistic regression analysis are given in Table 3. In the multivariable analysis, the odds for treatment seeking of HT increased with age and was highest among the age groups 75 years and older (among men AOR: 2.18; 95% CI 1.44–3.31 and among women AOR: 2.27; 95% CI 1.68–3.06). In men, education was not significantly associated with treatment seeking, but in women, if they had a secondary level education, they were more likely to take treatment compared to less educated women. In both men and women, if they were overweight/obese, their chances of taking treatment increased. Those with diabetes had higher odds of treatment seeking (among men AOR: 1.84; 95% CI 1.45–2.33 and among women AOR: 1.92; 95% CI 1.55–2.38). Men suffering from stroke were significantly associated with treatment seeking.

**Table 3:** Logistic regression results for currently taking treatment of hypertension among older adults, India, LASI, 2017–18.

	**AOR (overall)**	**95% CI**	**AOR (men)**	**95% CI**	**AOR (women)**	**95% CI**
**Individual factors**						
**Age groups**						
45–54	Ref.		Ref.		Ref.	
55–64	1.65^***^	(1.42, 1.93)	1.94^***^	(1.46, 2.57)	1.52^***^	(1.27, 1.81)
65–74	1.96^***^	(1.64, 2.34)	2.14^***^	(1.59, 2.86)	1.82^***^	(1.44, 2.29)
75+	2.24^***^	(1.77, 2.85)	2.18^***^	(1.44, 3.31)	2.27^***^	(1.68, 3.06)
**Education level**						
No education	Ref.		Ref.		Ref.	
Primary	1.11	(0.95, 1.29)	1.08	(0.84, 1.37)	1.14	(0.93, 1.40)
Secondary	1.30^*^	(1.05, 1.60)	1.17	(0.87, 1.57)	1.56^**^	(1.19, 2.04)
Higher	1.32^*^	(1.05, 1.67)	1.37	(0.98, 1.91)	1.16	(0.78, 1.73)
**Working status**						
Never worked	Ref.		Ref.		Ref.	
Currently working	0.78^**^	(0.65, 0.93)	0.66	(0.40, 1.10)	0.89	(0.72, 1.10)
Not currently working	0.95	(0.81, 1.11)	0.98	(0.61, 1.58)	0.91	(0.76, 1.10)
**Marital status**						
Currently married	Ref.		Ref.		Ref.	
Widowed	1.22^**^	(1.05, 1.41)	0.99	(0.70, 1.40)	1.25^*^	(1.05, 1.49)
D/S/D/Others^a^	0.76	(0.52, 1.10)	0.63	(0.33, 1.23)	0.84	(0.54, 1.30)
**BMI categories**						
Normal	Ref.		Ref.		Ref.	
Underweight	0.62^***^	(0.53, 0.73)	0.46^***^	(0.36, 0.59)	0.76^*^	(0.62, 0.94)
Overweight/obese	1.61^***^	(1.40, 1.84)	1.50^***^	(1.19, 1.90)	1.68^***^	(1.43, 1.99)
**Comorbidities**						
**Diabetes**						
No	Ref.		Ref.		Ref.	
Yes	1.88^***^	(1.60, 2.21)	1.84^***^	(1.45, 2.33)	1.92^***^	(1.55, 2.38)
**Stroke**						
No	Ref.		Ref.		Ref.	
Yes	1.85^***^	(1.34, 2.55)	2.12^**^	(1.33, 3.38)	1.53	(0.98, 2.41)
**Arthritis**						
No	Ref.		Ref.		Ref.	
Yes	0.99	(0.80, 1.23)	1.13	(0.81, 1.57)	0.94	(0.72, 1.22)
**Difficulty in ADL** ^b^						
No	Ref.		Ref.		Ref.	
Yes	1.20	(0.99, 1.47)	1.15	(0.80, 1.66)	1.23	(1.00, 1.51)
**Difficulty in IADL^c^**						
No	Ref.		Ref.		Ref.	
Yes	0.98	(0.85, 1.13)	0.87	(0.68, 1.10)	1.04	(0.88, 1.23)
**Lifestyle factors**						
**Moderate activities**						
Inactive	Ref.		Ref.		Ref.	
Active	0.85^*^	(0.74, 0.96)	0.92	(0.74, 1.16)	0.80^**^	(0.67, 0.94)
**Vigorous activities**						
Inactive	Ref.		Ref.		Ref.	
Active	0.94	(0.81, 1.10)	0.94	(0.75, 1.18)	0.93	(0.76, 1.13)
**Smoking tobacco**						
Never	Ref.		Ref.		Ref.	
Former	1.25	(0.96, 1.64)	1.50^*^	(1.09, 2.07)	0.71	(0.44, 1.14)
Current	0.98	(0.81, 1.19)	1.04	(0.81, 1.32)	0.92	(0.65, 1.30)
**Chewing tobacco**						
Never	Ref.		Ref.		Ref.	
Former	0.83	(0.58, 1.19)	0.73	(0.47, 1.14)	1.00	(0.56, 1.78)
Current	0.93	(0.79, 1.08)	0.80^*^	(0.64, 1.00)	1.07	(0.86, 1.34)
**Alcohol consumption**						
No	Ref.		Ref.		Ref.	
Yes	0.76^**^	(0.64, 0.91)	0.79^*^	(0.64, 0.98)	0.75	(0.49, 1.17)
**Household factors**						
**MPCE quintile**						
Poorest	Ref.		Ref.		Ref.	
Poorer	1.39^**^	(1.13, 1.71)	1.47^*^	(1.03, 2.11)	1.36^*^	(1.07, 1.73)
Middle	1.51^***^	(1.23, 1.87)	1.37	(0.96, 1.96)	1.63^***^	(1.29, 2.07)
Richer	1.63^***^	(1.32, 2.02)	1.66^**^	(1.16, 2.39)	1.63^***^	(1.29, 2.06)
Richest	1.81^***^	(1.45, 2.27)	1.96^***^	(1.34, 2.85)	1.73^***^	(1.35, 2.23)
**Religion**						
Hindu	Ref.		Ref.		Ref.	
Muslim	1.17	(0.97, 1.41)	1.40^*^	(1.01, 1.94)	1.05	(0.83, 1.33)
Christian	1.08	(0.82, 1.42)	1.02	(0.67, 1.56)	1.12	(0.78, 1.61)
Others^$^	1.24	(0.96, 1.60)	1.38	(0.97, 1.96)	1.19	(0.83, 1.69)
**Caste**						
Scheduled caste	Ref.		Ref.		Ref.	
Scheduled tribe	0.80	(0.62, 1.03)	0.76	(0.51, 1.14)	0.84	(0.61, 1.16)
OBC^#^	0.92	(0.77, 1.09)	0.88	(0.68, 1.14)	0.98	(0.78, 1.22)
Others	1.03	(0.86, 1.24)	1.00	(0.76, 1.32)	1.08	(0.85, 1.37)
**Place of residence**						
Rural	Ref.		Ref.		Ref.	
Urban	1.47^***^	(1.28, 1.69)	1.41^**^	(1.12, 1.79)	1.50^***^	(1.26, 1.78)
**Region**						
North	Ref.		Ref.		Ref.	
Central	0.95	(0.79, 1.15)	0.95	(0.69, 1.31)	0.94	(0.75, 1.20)
East	1.13	(0.95, 1.34)	1.18	(0.89, 1.55)	1.10	(0.88, 1.36)
Northeast	1.40^**^	(1.12, 1.74)	1.41^*^	(1.01, 1.97)	1.30	(0.97, 1.74)
West	1.99^***^	(1.58, 2.51)	2.21^***^	(1.55, 3.13)	1.84^***^	(1.37, 2.49)
South	2.77^***^	(2.27, 3.39)	2.73^***^	(1.96, 3.81)	2.71^***^	(2.12, 3.45)

# *Other Backward Classes*.

$ *Includes Sikh, Buddhist/neo-Buddhist, Jain, Parsi/Zoroastrian and others*.

a *Divorced, separated, and deserted*.

b *Activities of daily living includes dressing, walking across a room, bathing, eating difficulties, getting in or out of bed and toilet use (any one or more)*.

c *Instrumental Activities of Daily Living (IADL) includes preparing a hot meal, shopping for groceries, making telephone calls, taking medications, doing work around the house or garden, managing money and getting around or finding address in unfamiliar place (any one or more)*.

* *p < 0.05*,

** *p < 0.01*,

*** *p < 0.001*.

*BMI, Body Mass Index; AOR, Adjusted Odds Ratio; CI, Confidence Interval*.

Table 4 depicts the state-wise performance of self-reported HT, undiagnosed HT (newly diagnosed cases or measured at the time of survey), total prevalence (addition of self-reported HT and undiagnosed HT), currently taking medicine (treated), and controlled HT (on treatment and had a normal BP). At the national level, 27.4% of individuals had self-reported HT and 17.8% were not aware about their HT condition and learned of it at the time of survey. This indicates that about four out of 10 adults aged 45 years and older are suffering from HT and only 60% are aware of their hypertensive status. Among hypertensive individuals, 73% reported currently taking treatment, and only 10.4% had a normal BP. Table 4 also shows considerable variation among states and UTs in the proportion of all these indicators, as total HT was highest in Lakshadweep (66.1%) and lowest in Uttar Pradesh (34.7%) and Mizoram (34.8%). Undiagnosed HT cases varied from 9.7% in Jammu and Kashmir to 38.5% in Nagaland and 28.6% in Chhattisgarh; and those diagnosed out of total HT cases varied from 29.2% in Nagaland and 36.6% in Chhattisgarh to 80.7% in Jammu and Kashmir; treated HT cases ranged from 31.7% in Arunachal Pradesh to 94.7% in Goa; and controlled BP varied from 1.1% in Nagaland to 23.5% in Goa. It is important to note that Jammu and Kashmir, Chandigarh, Haryana and Goa performed better in comparison to other states as the proportion of self-reported HT out of total HT was higher, indicating better performance of the health systems in these states.

**Table 4:** State-wise prevalence of self-reported, undiagnosed, overall, and controlled hypertension among older adults in India, LASI, 2017–18.

**States**	**Self-reported HT**	**Total prevalence**	**Gap (undiagnosed HT)**	**Performance of state (diagnosis)**	**Currently taking medicine**	**Controlled HT**
	***a***	***b***	***c*** **=** ***b*****-*****a***	***d*** **=** a*100/***b***	**(***e***)**	**(***f***)**
Jammu and Kashmir	40.6	50.3	9.7	80.7	85.3	16.5
Himachal Pradesh	32.9	56.1	23.2	58.6	63.7	8.4
Punjab	42.8	62.1	19.2	69.0	73.9	14.5
Chandigarh	39.6	53.5	14.0	73.9	80.7	19.6
Uttarakhand	26.6	48.1	21.5	55.3	56.6	8.4
Haryana	38.5	52.5	14.1	73.2	55.2	12.5
Delhi	35.8	52.5	16.7	68.2	68.2	11.7
Rajasthan	27.3	42.3	15.0	64.5	59.7	9.6
Uttar Pradesh	20.0	34.7	14.7	57.7	57.5	7.0
Bihar	25.1	42.1	17.1	59.5	49.2	6.5
Arunachal Pradesh	22.6	44.8	22.2	50.4	31.7	2.4
Nagaland	15.8	54.3	38.5	29.2	60.5	1.1
Manipur	28.7	45.7	17.0	62.8	69.9	10.2
Mizoram	24.0	34.8	10.8	69.0	43.7	4.7
Tripura	30.4	48.2	17.8	63.1	68.0	10.8
Meghalaya	25.9	50.0	24.0	51.9	78.1	9.5
Assam	31.1	48.1	17.0	64.6	64.7	7.9
West Bengal	29.6	44.4	14.9	66.5	74.6	9.5
Jharkhand	21.7	43.3	21.6	50.1	64.1	6.3
Odisha	20.4	38.1	17.8	53.5	67.7	7.8
Chhattisgarh	16.5	45.1	28.6	36.6	68.8	5.7
Madhya Pradesh	20.0	38.4	18.4	52.1	64.2	7.2
Gujarat	25.7	46.5	20.8	55.3	69.2	10.1
Daman and Diu	32.9	52.6	19.7	62.6	79.8	13.0
Dadra and Nagar Haveli	17.0	40.8	23.8	41.7	69.4	6.6
Maharashtra	28.9	49.1	20.2	58.8	86.4	14.1
Andhra Pradesh	35.0	53.0	18.0	66.0	88.4	15.2
Karnataka	32.7	51.1	18.4	64.0	91.7	15.6
Goa	44.1	57.9	13.8	76.2	94.7	23.5
Lakshadweep	35.5	66.1	30.6	53.7	76.7	9.4
Kerala	41.0	60.3	19.3	68.0	87.6	18.0
Tamil Nadu	26.3	45.2	18.8	58.3	76.7	10.4
Puducherry	32.7	49.3	16.6	66.3	87.8	18.1
Andaman and Nicobar Island	41.2	64.8	23.7	63.5	78.8	10.8
Telangana	31.0	47.3	16.3	65.5	87.6	15.4
**Total**	**27.4**	**45.2**	**17.8**	**60.6**	**73.0**	**10.4**

**Figure 1:**
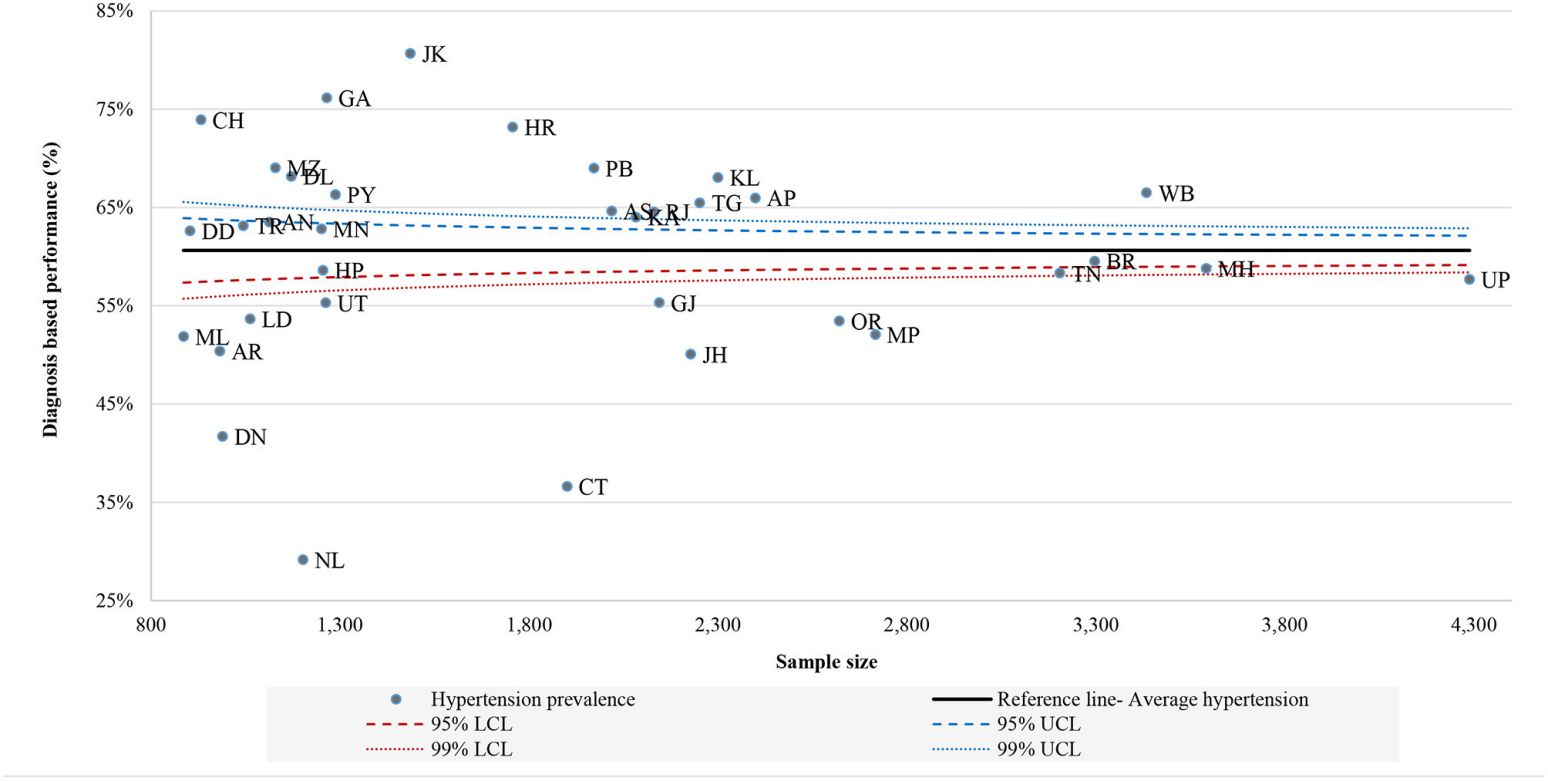
Funnel plot of performance of states: diagnosis of hypertension, LASI (2017–18).

**Figure 2:**
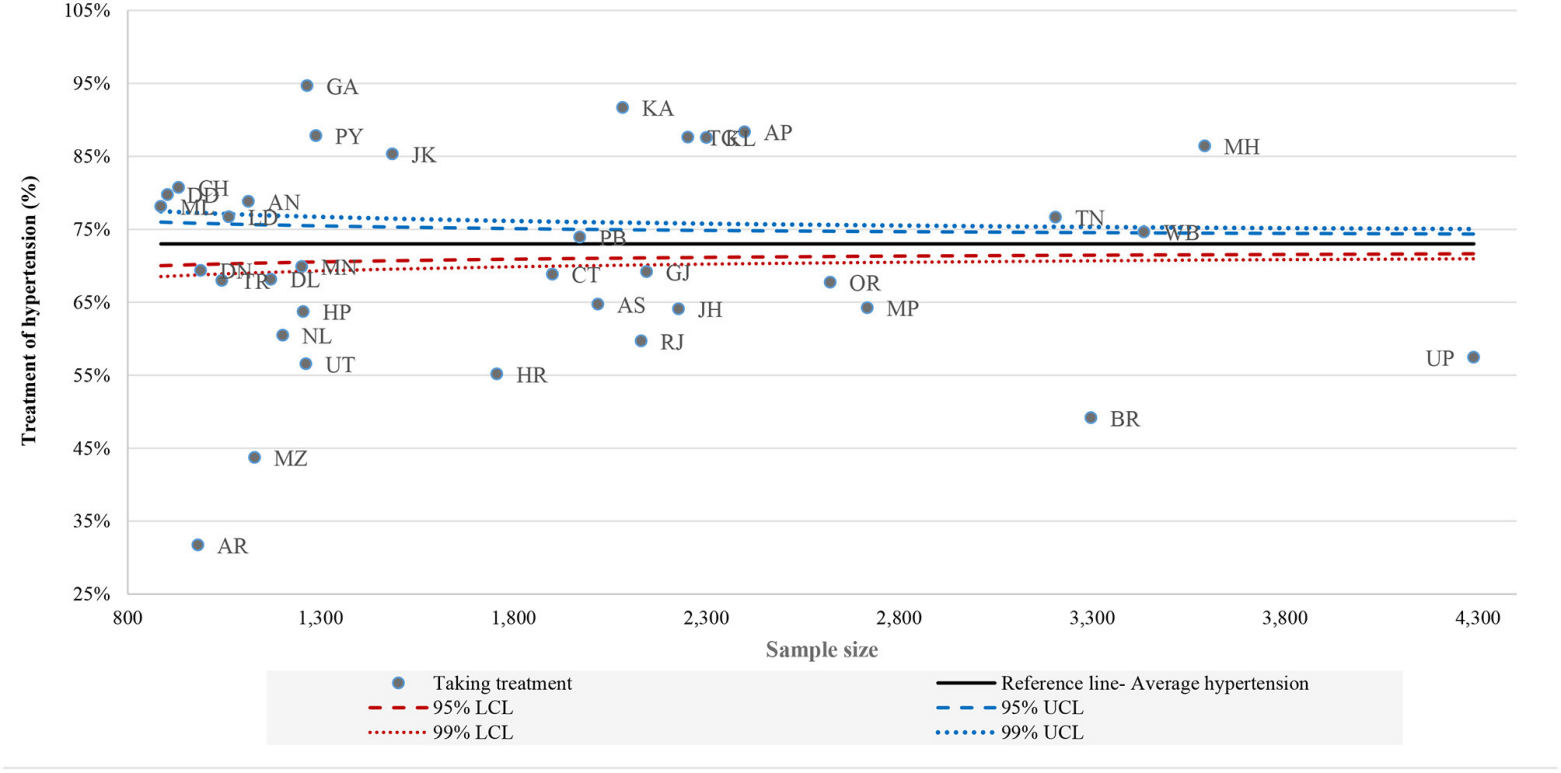
Funnel plot of performance of states: treatment of hypertension, LASI (2017–18).

**Figure 3:**
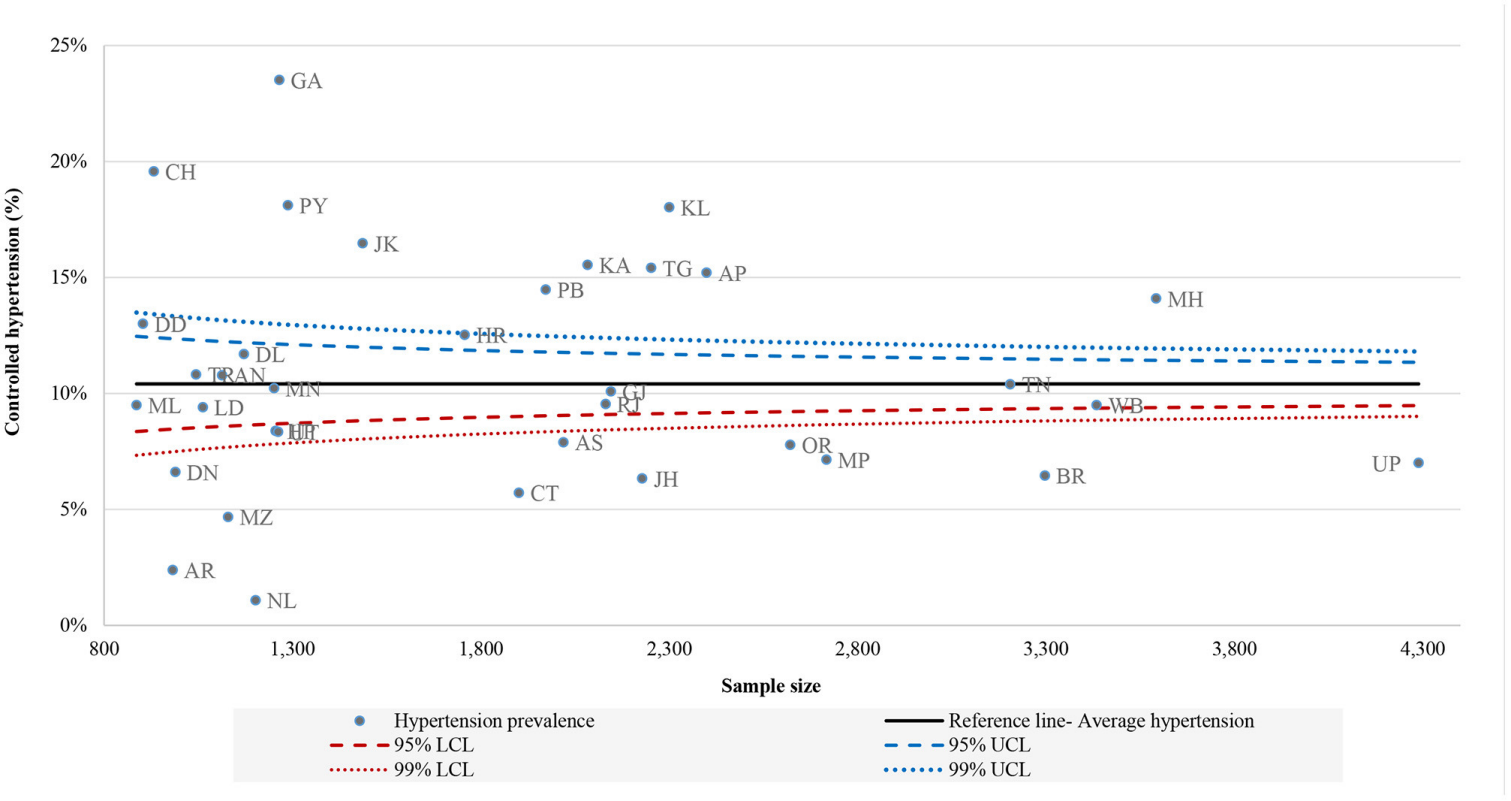
Funnel plot of performance of states: controlled hypertension, LASI (2017–18).

Figures 1–3 shows the funnel plots of state performance with respect to diagnosis, treatment, and control of HT. The figures show the states with the lowest prevalence of diagnosed HT, lowest proportion of patients taking treatment, and lowest percentages of controlled HT with the highest percentage of these indicators compared with Indian average figures, as indicated by a solid line parallel to the x-axis. The prevalence of these HT-related indicators at the national level was used as a baseline comparison for each state. Data points closer to the y-axis are states with a smaller population size and those on the right side have larger population size. Data points that are outside the confidence interval (CI) band are pointed out as having a different prevalence of HT-related indicators from the Indian average. Those states outside the 99% CI can be measured as outliers in terms of their performance with respect to the mentioned indicators. States that are above the Indian average are the best-performing states and those below the national average are the worst-performing states in terms of awareness, treatment and controlled HT. Health system performance in Jammu and Kashmir, Goa and states mainly from the northern region were far better than other states, since out of total HT caseload, more than 70% of cases were diagnosed. Conversely, Nagaland, Chhattisgarh and Dadra and Nagar Haveli were the states where only <45% people are aware of their HT condition. Figure 2 shows variation in treatment seeking for HT, where most of the states in the south and west performed better than east and central regions. This low treatment seeking could be due to unaffordable medication, lack of availability and the accessibility of health centres. Similarly, Figure 3 illustrates the considerable variation in state performance with respect to controlled HT. Of all the states and UTs, 11 states, namely Uttar Pradesh, Bihar, Odisha, Madhya Pradesh, Jharkhand, Assam, Chandigarh, Nagaland, Arunachal Pradesh, Mizoram and Damn and Diu performed so poorly with respect to controlled HT that they were below the lower limits of the distribution of the funnel plot, which was created at the 99% confidence bands. Mainly 10 states from the southern, eastern and western regions were above the overall Indian baseline at the 99% band.

Finally, we also attempt to understand the factors that explain the variation in state performance as observed in the funnel plots. The regression results for self-reported HT (Model 1) and treatment (Model 2) are presented in Table 5. The results suggest that having a higher proportion of the population below the poverty line was significantly related to lower HT awareness (*p* = 0.012). Total OOPE was significantly associated with an increase in self-reported HT (*p* = 0.043). The literacy rate was positively but not significantly related to HT awareness (*p* = 0.313). Regarding treatment seeking behaviour in Model 2, higher literacy rates (*p* = 0.059) and greater availability of specialist doctors (*p* = 0.061) at CHCs significantly increased the prevalence of treatment for HT at a 10% level of significance.

**Table 5:** Regression results for state performance- self-reported HT and its treatment, 2017–18.

**Variables**	**Self-reported hypertension (model-1)**			**Treatment for hypertension (model-2)**		
	**Coeff**.	***P*** **-value**	**95% CI**	**Coeff**.	***P*** **-value**	**95% CI**
HDI	6.422	0.842	(−59.23, 72.07)	71.035	0.292	(−65.08, 207.15)
% Population BPL	−0.329	0.012	(−0.579, −0.08)	−0.215	0.398	(−0.732, 0.302)
Literacy rate	0.176	0.313	(−0.177, 0.53)	0.704	0.059	(−0.029, 1.436)
Doctors available at PHCs	0.000	0.918	(−0.005, 0.004)	−0.006	0.216	(−0.015, 0.004)
Specialists available at CHCs	−0.003	0.836	(−0.028, 0.023)	0.051	0.061	(−0.002, 0.104)
OOPE	0.017	0.043	(0.001, 0.033)	−0.001	0.958	(−0.035, 0.033)
Per capita health expenditure	−0.001	0.585	(−0.003, 0.001)	−0.005	0.029	(−0.009, −0.001)
*N*	32			32		
*R* ^2^	57.5			43.7		

*HT, Hypertension; Coeff., Coefficient; HDI, Human Development Index; BPL, Below Poverty Line; PHC, Public Health Centre; CHC, Community Health Centre; OOPE, Out-of-Pocket expenditure (includes inpatient and outpatient expenditures)*.

## Discussion

India is a diverse country with considerable variations in terms of socio-economic development, caste, and cultural practises among its population. With increasing urbanisation, improved standards of living due to economic growth, associated lifestyle changes and an increasingly ageing population as a result of increased life expectancy, India is fertile ground for an increasing prevalence of NCDs. In addition, a health transition both in terms of demographic and epidemiological transition is rapidly taking place in India, with a shift from a predominantly young population to an increasingly ageing population, and from high morbidity and mortality due to acute, infectious and communicable diseases in the younger population to chronic non-communicable diseases in the elderly population. For example, disability-adjusted life-years (DALYs) due to NCDs has increased from 29.2% in 1990 to 57.9% in 2019 (3). Similarly, the Global Burden of Diseases study estimates that DALYs attributed to HT almost doubled from 18 to 37 million in the period from 1990 to 2019 (3). However, in spite of the increasing prevalence of HT, there has been no significant improvement in the diagnosis, treatment and control of HT over the years (22).

As healthcare is a state responsibility in India, for effective targeting of health services at the local level, sub-national level planners and policy makers must have a reliable estimate of not only the overall prevalence of HT, but also its distribution and the characteristics of the sub-groups of the population that are aware of their hypertensive status and are taking treatment. In addition, understanding the variation in state performance with respect to the diagnosis and treatment of HT at the sub-national level is necessary for planning effective strategies to control HT.

Our study findings confirm that the overall prevalence of HT among older adults over the age of 45 years is 45.2% (4 in 10) with significant variation among states. Among the hypertensive participants, only 60.6% (6 in 10) were aware of their condition. Although 73.0% (7 in 10) of these participants who were aware of their diagnosis were currently taking treatment at the time of the survey, only 10.4% (1 in 10) had adequately controlled their HT. Comparing our findings with other countries shows that our estimated HT prevalence is lower than that of China (26) and many developed countries such as the United States (27), Ireland and the Russian Federation (28), but higher than that in many other neighbouring countries (29–31). For example, in a systematic review based on 33 observational studies from seven South Asian countries, the overall prevalence of HT was nearly 27%, ranging from 17.9% in Bangladesh to 33.8% in Nepal (32). Our higher prevalence rate of 45% compared to the South Asian study mentioned above and others can to a large extent be explained by the selection of participants who were above the age of 45 years in our study, whereas other studies considered adults over the age of 18 years.

A study based on multiple national-level surveys on the overall prevalence of HT among older adults aged 50 years and above in select high-income countries (HICs), upper-middle-income countries (UMICs) and lower-middle-income countries (LMICs), estimated the rates of awareness, treatment and control of HT from 78.0, 67.9, and 29.8% in HICs, to 40.3, 31.6, and 7.3% in UMICs, and 43.7, 24.2, and 12.5% in LMICs (28). In comparison, the rates of awareness, treatment and control of HT in our study were 60.6, 44.0, and 10.4%, respectively, with considerable variation among states. There may be number of factors contributing to lack of awareness of HT in states like Chhattisgarh, Bihar, Jharkhand, Madhya Pradesh, Odisha, Himachal Pradesh, Gujarat and Uttarakhand. HT is usually asymptomatic, so many individuals who are hypertensive may not recognise this condition and thus may not be aware of it, may not come in contact with doctors/health facilities, may provide inadequate/incomplete information to doctors or their blood pressure measurements may not have been measured in spite of coming into contact with a doctor/health facility. This represents a missed opportunity in terms of diagnosis, or even after being diagnosed as hypertensive, survey participants may not have remembered at the time of the survey (recall bias). However, it appears that the main reason could be due to issues around access and availability of health facilities and a lack of screening programmes, such that fewer individuals have the opportunity to measure their blood pressure.

As cheap and effective drugs are available for the treatment and control of HT, and the consequences of not treating HT are associated with significant adverse health outcomes, it is unacceptable that India has such low treatment and control rates. Issues around financial barriers resulting in a lack of affordability to purchase anti-hypertensive drugs from the pharmacy and lack of consistent availability of these drugs in the public sector may be important reasons for low treatment rates in states like Rajasthan, Uttar Pradesh, Odisha, Assam, Manipur and Uttarakhand. It is even more of a concern as only 37.2% of India's population is covered under any health insurance (25).

It is also interesting to note that, although the treatment rate of those currently taking medication is similar to that of India, Pakistan has a higher control rate of 22.3% (33). Similarly, our overall treatment rate for all hypertensives was 44% and comparable to Bangladesh at 43%, yet Bangladesh has a much better HT control rate at 22% (34) when compared to our 10% control rate. India's poor performance with respect to HT control rate raises issue around the treatment protocol, adherence regimes and affordability, monitoring and follow-up. Further research may be undertaken to understand the underlying causes for such poor HT control rates in India.

Given poor awareness and low treatment and control rates for HT as per our study findings, it therefore raises the question of the effectiveness of the national CVD control programme and national NCD programme. For instance, the National Programme for Prevention and Control of Cancer, Diabetes, Cardiovascular Diseases and Stroke (NPCDCS) was launched in 2010 with the aim of preventing and controlling NCDs through awareness, lifestyle changes and early diagnosis of high risk individuals. However, only 4 million persons attended NCD clinics and were screened for HT in 2018 (25). Our findings in terms of low rates of the treatment and control of HT are similar to other studies in India and elsewhere and raise concerns regarding impending cardiovascular mortality and morbidity (13, 35, 36). The literature suggests that there are significant barriers in terms of access and utilisation of diagnostic services and therefore treatment of HT in India. In a country like India, where OOPE constitute 70% of total health spending (37), financial barriers can be a significant concern as both diagnosis and the purchase of hypertensive drugs may require OOPE by the majority of the population. This is further exacerbated as diagnostic services, doctor consultations or admissions in the private sector are all on a fee-for-service basis. There is evidence to suggest that high OOPE for health care contributes to impoverishment in India (38).

India is home to 17.7% of the world's population and contributes 20% of the global burden of diseases due to NCDs (39). Currently, 60% of hospitals, 75% of dispensaries, and 80% of doctors are in the urban areas serving only 28% of the country's population (40). Whereas, the majority of India's population resides in rural areas where decades of underfunding have resulted in a weak public health care system with inadequate health infrastructure, lack of adequate human resources for health and low availability of drugs, resulting in significant barriers to accessing health services. In addition, the population residing in rural areas has other unfavourable social determinants of health like lower literacy rates and lower socio-economic conditions that further prevent the effective implementation of preventive and promotive health programmes.

Our findings suggest that even more developed states like Karnataka and Kerala have an 11 and 17% shortage of doctors in PHCs and a 67 and 80% shortage of specialist doctors in CHCs in rural areas, respectively. For India as a whole, there is a shortfall of over 78% of specialist doctors at CHCs in rural areas (41). A study published in the Lancet confirms our findings that 83% of surgeon and physician roles are vacant in India's rural areas (42). Similarly, a 72% shortfall has been observed with respect to health assistants (HA) at the PHC level. Although unacceptable, the situation is comparatively better in urban areas than in rural areas. There is a 46% shortfall of auxiliary nurse midwives (ANMs) in urban PHCs, who are a key workers in a number of public health programmes. In addition, there is shortfall of staff including doctors, specialists, nurses, pharmacists and technicians in urban PHCs and CHCs. Thus, it appears that unless large investments are considered, India's existing public health infrastructure will be unable to cope with the epidemic of NCDs. Unfortunately, the latest budget was a missed opportunity to remedy the situation (43). It is therefore imperative that the meagre government health spending of 1.8% of GDP is raised significantly to improve the public health infrastructure, staffing levels and equipment, and availability of drugs in general, and in particular with respect to screening, diagnostic services, treatment and the management of HT.

Policy makers will have to ensure that this variation among states and sub-groups is minimised and that public health care systems are improved, especially in underperforming states. Lessons could be learnt from well-performing states like Goa, Kerala, Punjab, Karnataka and Chandigarh. A number of reasons can be hypothesised for good performing states in terms of the diagnosis and treatment of HT, including higher HDI, higher literacy rates, a strong public health sector including the primary health care network, and better access to quality health services including the availability of human resources and drugs as compared to underperforming states.

Given the extent of variation in state performance, a one-size-fits-all approach to reducing HT across India may not be an effective strategy. Policy makers may rather adopt a flexible approach depending on a state's development and its performance in terms of the diagnosis and treatment of HT. Policy makers may consider targeting underperforming states as identified by the funnel plot and thus attempt to minimise the variation in performance across the states of India. As per our findings, states with a large population, high prevalence of HT and low performance in terms of the diagnosis and treatment of HT can be identified as high impact states and should be given priority by policy makers, as these states have significant potential for reducing the avoidable mortality and morbidity associated with HT and its consequences. It is proposed that selective targeting of high risk individuals may be adopted as a strategy in states like West Bengal where the prevalence of HT is low but state performance in terms of diagnosis and treatment rate is high. Conversely, states like Himachal Pradesh, Uttarakhand, and Gujarat where the prevalence of HT is high and state performance is low in terms of diagnosis and treatment of HT would benefit from a rapid scale-up of primary level of interventions at a population level.

More generally, India could adopt multipronged strategies that include improved screening and measurement of blood pressure for high risk individuals, health education programmes and free availability of hypertensive drugs in order to improve the diagnosis and treatment rates. Medical staff should be trained to ensure that every contact with the health staff involves opportunistic screening for HT so that appropriate treatment can be initiated at the earliest. Besides opportunistic screening at health facilities, screening should also be undertaken at the community level, especially in high risk states. Given the issues around the affordability of private doctors and the lack of doctors in the public health sector, community health workers could be trained in screening those at risk and referring individuals for further management. Studies have shown that community health workers are effective in a number of public health programmes in various settings (44, 45). In addition, mass screening camps for the early detection of HT cases can be considered in high risk areas with limited health facilities. Such a strategy, based on the primary care level with an emphasis on early diagnosis and prompt treatment of HT, is likely to be highly cost-effective as the economic burden of untreated HT to the individual and the health system can be enormous.

Our study had potential strengths and limitations. The study's main strength was the large sample size and national-level representation of the Indian older adults. Moreover, the present study contributes to the existing literature by not only providing current estimates of the prevalence, awareness, treatment and control of HT at sub-national levels but also provides estimates of performance of states with respect to the diagnosis and treatment of HT. Despite these strengths, all the limitations of cross-sectional survey data apply to this study as it is based on the first wave of the LASI data, thus fails to establish the causal relationship between the observed associations. It may also be noted that the Joint National Committee (JNC) 7 criteria for defining hypertension (i.e., self-reported HT, SBP ≥140 or DBP ≥90, and currently on medication) (46) was not used in this study. In the context of India, with number of barriers like literacy/awareness, access to health care services, and financial affordability to drugs and treatment, we believe that using the JCN-7 definition would underestimate the true prevalence of HT in the population. Moreover, we did not include the non-pharmacological treatments, dietary habits and life-style changes in the analysis that could impact on the treatment of HT.

Given India's population, its approach to reducing HT in its high impact states will determine the attainment of national NCD targets and global SDG targets.

## Data Availability Statement

Publicly available datasets were analysed in this study. This data can be found here: the study uses secondary data which is available on reasonable request through https://www.iipsindia.ac.in/content/lasi-wave-i.

## Ethics Statement

The studies involving human participants were reviewed and approved by the Indian Council of Medical Research (ICMR) extended the necessary guidelines and ethics approval for undertaking the LASI survey. The patients/participants provided their written informed consent to participate in this study.

## Author Contributions

MB conceptualised and designed this study. LKD, MK, and PD were involved in data analysis and statistical methods. All authors contributed to the drafting, reviewing, revising the manuscript, and seen and approved the final version of the manuscript.

## Funding

This research has received funding from the LSE Covid Impact Fund for Research and Knowledge Exchange.

## Conflict of Interest

The authors declare that the research was conducted in the absence of any commercial or financial relationships that could be construed as a potential conflict of interest.

## Publisher's Note

All claims expressed in this article are solely those of the authors and do not necessarily represent those of their affiliated organizations, or those of the publisher, the editors and the reviewers. Any product that may be evaluated in this article, or claim that may be made by its manufacturer, is not guaranteed or endorsed by the publisher.
